# Patients with Coronary Artery Disease Have Lower Levels of Antibody to Heat-Stressed Fibroblast Derived Proteins, versus Normal Subjects

**DOI:** 10.1155/2021/5577218

**Published:** 2021-06-17

**Authors:** Azin Aghamajidi, Hesam Babaei Khameneh, Narges Amirjamshidi, Seyed Farzad Jalali, Haleh Akhavan-Niaki, Soraya Khafri, Seyedeh Narges Mousavi, Monireh Golpour, Maryam Mehri, AmrollahMostafazadeh

**Affiliations:** ^1^Students Research Committee, School of Medicine, Babol University of Medical Sciences, Babol, Iran; ^2^Department of Immunology, School of Medicine, Iran University of Medical Sciences, Tehran, Iran; ^3^Department of Cardiology, Ayatollah Rouhani Hospital, Babol University of Medical Sciences, Babol, Iran; ^4^Cellular and Molecular Biology Research Center, Health Research Institute, Babol University of Medical Sciences, Babol, Iran; ^5^Department of Social Medicine, Babol University of Medical Sciences, Babol, Iran; ^6^Molecular and Cell Biology Research Center, Student Research Committee, Faculty of Medicine, Mazandaran University of Medical Science, Sari, Iran

## Abstract

Cellular stress response plays an important role in the pathophysiology of coronary artery disease (CAD). Inhibition of cellular stress may provide a novel clinical approach regarding the diagnosis and treatment of CAD. Fibroblasts constitute 60-70% of cardiac cells and have a crucial role in cardiovascular function. Hence, the aim of this study was to show a potential therapeutic application of proteins derived from heat-stressed fibroblast in CAD patients. Fibroblasts were isolated from the foreskin and cultured under heat stress conditions. Surprisingly, 1.06% of the cells exhibited a necrotic death pattern. Furthermore, heat-stressed fibroblasts produced higher level of total proteins than control cells. In SDS-PAGE analysis, a 70 kDa protein band was observed in stressed cell culture supernatants which appeared as two acidic spots with close pI in the two-dimensional electrophoresis. To evaluate the immunogenic properties of fibroblast-derived heat shock proteins (HSPs), the serum immunoglobulin-G (IgG) was measured by ELISA in 50 CAD patients and 50 normal subjects who had been diagnosed through angiography. Interestingly, the level of anti-HSP antibody was significantly higher in non-CAD individuals in comparison with the patient's group (*p* < 0.05). The odds ratio for CAD was 5.06 (95%CI = 2.15‐11.91) in cut-off value of 30 AU/mL of anti-HSP antibody. Moreover, ROC analysis showed that anti-HSP antibodies had a specificity of 74% and a sensitivity of 64%, which is almost equal to 66% sensitivity of exercise stress test (EST) as a CAD diagnostic method. These data revealed that fibroblast-derived HSPs are suitable for the diagnosis and management of CAD through antibody production.

## 1. Introduction

Coronary artery disease (CAD) which mainly occurs due to atherosclerosis is the leading cause of death worldwide [1]. Atherosclerosis can be viewed as bidirectional stress for cardiac cells from the periphery to the heart and vice versa. Different types of endogenous and exogenous stressors contribute to the pathophysiology of atherosclerotic CAD [2]. Metabolic syndrome, genetic variations, and smoking are the major risk factors for atherosclerosis through the induction of oxidative stress [3–5]. Moreover, environmental stimuli such as solar UV radiation have been considered a possible detrimental factor that may activate oxidative stress by the generation of reactive oxygen species (ROS) [6]. Oxidative stress and ROS production are highly associated with atherosclerotic plaque formation through biochemical modification of low-density lipoprotein (LDL) [7]. Based on the evidence, subsequent chronic inflammatory responses play a critical role in the development of atherosclerosis [8–10]. Besides, inflammatory cytokines including interleukin-1*β* (IL-1*β*), IL-6, IL-12, and tumor necrosis factor-alpha (TNF-*α*) accelerate atherosclerosis progression [11]. This primary stress originates from peripheral tissues which might exert deleterious effects on different types of cells, especially cardiac cells. The partial or complete stenosis of coronary arteries results in restricted blood flow as well as reduced left ventricular ejection fraction (LVEF) [12, 13]. Vascular occlusion may trigger the second cascade of cellular stress due to the hypoxia and nutrient deficiency which is occurred in the heart and peripheral tissues. Principally, diverse forms of cellular stress such as heat shock and nutrient/serum starvation elicit an intracellular signaling network recognized as an integrated stress response (ISR) in eukaryotic cells [14]. The ISR includes a global reduction of protein synthesis as well as upregulation of specific genes such as heat shock proteins (HSPs) that play a pivotal role in cell survival during stress conditions [15]. HSPs trigger immune activation which is readily traceable by screening the specific antibodies in blood circulation [16]. Endoplasmic reticulum (ER) stress is one of the primary ISR inducers in different cells including coronary artery endothelial cells, smooth muscle cells (SMCs), and macrophages that play a key role in atherosclerosis [17]. Furthermore, there is an increasing interest in the investigation of the role of fibroblasts, as another cardiac cell, in atherosclerosis. Fibroblasts are ubiquitous cells that exist almost in all peripheral tissues and organs particularly the skin which could sense chemical and physical stress [18]. Furthermore, fibroblasts constitute 60-70% of the cardiac cells and play an important role in the health or disease of the cardiovascular system [19, 20]. Also, fibroblasts account for about 33% of aortic cells in mice that play critical functions in tissue homeostasis [21]. In addition, different numbers of fibroblast clusters have been observed in both healthy and atherosclerotic tissues [21]. On the other hand, Golpour et al. have shown that serum starvation can significantly induce the production of more than 90 different proteins with cell survival and cell growth-promoting properties by human skin fibroblasts [22]. Besides, serum starvation can be considered a potential tool for immunoregulation through upregulation of transforming growth factor-beta (TGF-*β*) and CD4 + CD25 + FOXP3 + CD127-T-regulatory (Treg) cell [23]. These observations inspired a nuance idea implicating that heat shock and HSPs which direct the stressed cells toward an adaptation response, probably have some therapeutic applications. Hence, this article is aimed at evaluating the fibroblast response to heat shock that could provide new insights into a better understanding of atherosclerosis pathogenesis and treatment of the disease.

## 2. Materials and Methods

### 2.1. Subjects

**Figure 1:**
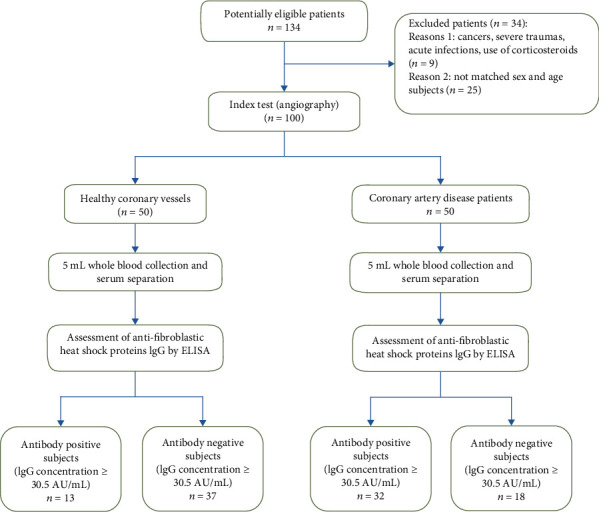
Overall study design flowchart. Among 134 potentially patients who referred for angiography, fifty patients with coronary atherosclerosis (stenosis ≥ 50%) and fifty healthy individuals (no plaque) were selected via the gold standard method. Serum was separated, and anti-HSP IgG was measured by ELISA assay. ^∗^NEG, POS, and con stand for negative, positive, and concentration, respectively.

Among 134 subjects with positive clinical signs and the abnormal result of routine tests (including chest pain, shortness of breath, positive radioisotope scan, abnormal exercise stress test (EST), and echocardiography) who were referred to the catheterization laboratory of Ayatollah Rouhani Hospital, Babol University of Medical Sciences, for definitive diagnosis by coronary angiography (Siemens, Germany), fifty patients with coronary atherosclerosis with more than 50% stenosis in at least one coronary artery (31 males, 19 females, mean age ± SD: 60.7 ± 10.39 years) were selected as the patient group. Also, fifty individuals without a plaque in coronary arteries were considered the healthy control group (21 males, 29 females, mean age ± SD: 62 ± 12.86 years). Thirty-four subjects were excluded from the study based on the criteria described in Figure 1. Then, whole blood was collected from both patient and control subjects; the serum was separated and stored at -20°C for further analysis. Hypertensive subjects (blood pressure ≥ 14 mmg) were determined by referring to their medical records. Written consent was obtained from each participant, and the research proposal was confirmed by the Research Ethics Committee of Babol University of Medical Sciences (MUBABOL.REC.1394.89).

### 2.2. Fibroblast Culture, Heat Shock, and Determination of Secreted Protein Concentration

Fibroblasts were isolated from the human foreskin based on the previously described protocol [24]. About 1.5 × 10^5^ fibroblasts at passages 3-8 were cultured in the T-25 flasks until 80% confluence. The flasks were incubated at 43°C for heat stress while one of them remained at 37°C as the control of the unstressed cells. After 2 h, the cell culture medium was removed from all flasks, and the cells were washed twice with PBS. Fresh serum-free Dulbecco's Modified Eagles Medium (DMEM) (BIOWEST, France) was added to flasks, and the cells were incubated for 6 h in standard cell culture conditions. Following the incubation time, cell culture supernatants were collected and concentrated using Amicon ultracentrifuge filter (cut off: 3 kDa) in 7500 × g for 40 min. Total protein concentration was determined by Bradford method [25]. The absorption of DMEM solution with Bradford reagent was subtracted from samples as the background color.

### 2.3. Flow Cytometry Analysis

After harvesting the cell culture supernatants, the heat-stressed fibroblasts were incubated overnight with DMEM + 10%FBS. Then, fibroblasts were detached by trypsin-EDTA and were prepared for analysis with flow cytometry (Partec, Germany) after staining with annexin V/propidium iodide (PI) according to the manufacturer's protocol of Annexin/PI (eBioscience, USA).

### 2.4. Sodium Dodecyl Sulfate Polyacrylamide Gel Electrophoresis (SDS-PAGE)

500 *μ*L of the concentrated samples which had been prepared from heat-stressed fibroblast culture was mixed with cold acetone as a 1 : 4 ratio and incubated overnight at -20°C. Then, the sample was centrifuged at 10000 × g at 4°C for 30 min. The precipitated proteins were dissolved in 20 *μ*L of distilled water, and the sample was loaded on 12% polyacrylamide gel. The electrophoresis procedure was run at 120 V for 3 h (Amersham, USA), and the gel was stained with silver nitrate. This experiment was repeated three times independently.

### 2.5. Two-Dimensional Electrophoresis (2DE)

20 *μ*g of protein prepared from heat-stressed fibroblasts was incubated overnight with 250 *μ*L of rehydration buffer (8 M urea, 4%m/v CHAPS, 65 mM DTT, 0.001%m/v Bromophenol blue). The protein molecules were focused using the following program by Ettan IPGphor III (GE Healthcare Life Sciences, USA) at 20°C, 50 V for 30 min, 100 V for 30 min, 150 V for 30 min, 250 V for 30 min, 1000 V gradient for 1 h, 8000 V gradient for 2 h, and 8000 V for 40,000 V/h. For protein reduction, the strip was incubated in equilibration buffer (0.05 M Tris–HCl, pH 8.8, 6 M urea, 30% glycerol, 2% *w*/*v* SDS) containing 130 mM DTT for 15 min, and alkylation process was done in a separate tube containing equilibration buffer with 135 mM iodoacetamide shaken for 15 min. Then, the strip was transferred to 12% SDS-PAGE gel, and the electrophoresis process was run. The gel was stained by Coomassie Blue R-250. This experiment was repeated two times independently.

### 2.6. Assessment of Serum Anti-HSP IgG with ELISA

10 *μ*g/mL of concentrated culture supernatant of heat-stressed fibroblast (standardized by Bradford method) was coated in 96-well SPL Maxisorp Immunoplates (China) overnight at 4°C. Then, the supernatant was removed, and the plate was blocked with 1% bovine serum albumin (BSA) in phosphate-buffered saline (PBS) for 4 h at room temperature (RT). After five times washing with rinsing buffer (1x PBS with 0.1% Tween 20), 100 *μ*L diluted serum sample (1 : 100 in 1x PBS, 1% BSA, and 0.1% Tween 20 assay diluent) and 100 *μ*L high positive anti-heat-stressed fibroblastic protein serum (pooled positive serum diluted in assay diluent solution in 1 : 2 ratio) were added to each well. Also, 100 *μ*L assay diluent solution was dispensed in five wells as the negative control. Then, the plates were incubated at RT for 1 h. After five times washing, 100 *μ*L horseradish peroxidase (HRP) conjugated anti-human IgG (1 : 4000 in assay diluent) was added to each well and incubated at RT for 1 h. After washing thoroughly and tapping, hydrogen peroxide (H2O2) and 3, 3′, 5, 5′-Tetra-Methyl Benzidine (TMB) solution was added. The stop solution (1M H2SO4) was dispensed after color development, and the optical density (OD) of samples was measured with an ELISA reader (Rayto, China) at 450 nm. The antibody concentration was obtained based on a standard curve plotted according to OD values of high positive pooled serum for fibroblastic HSPs, which was expressed as AU/mL. This experiment was repeated two times independently.

### 2.7. Statistical Analysis

Data are expressed as mean ± SD and median. The normal distribution of data was examined by the Kolmogorov-Smirnov test. Mann–Whitney *U* test and logistic regression were used to compare the level of specific anti-HSP antibody and odds ratio in the adjustment of demographic variables, respectively. All data were analyzed by the SPSS 22 software. *p* value < 0.05 was statistically considered as the significance level.

## 3. Results

### 3.1. Skin Fibroblasts Secrete Two Acidic Proteins in Response to Heat Shock

**Figure 2:**
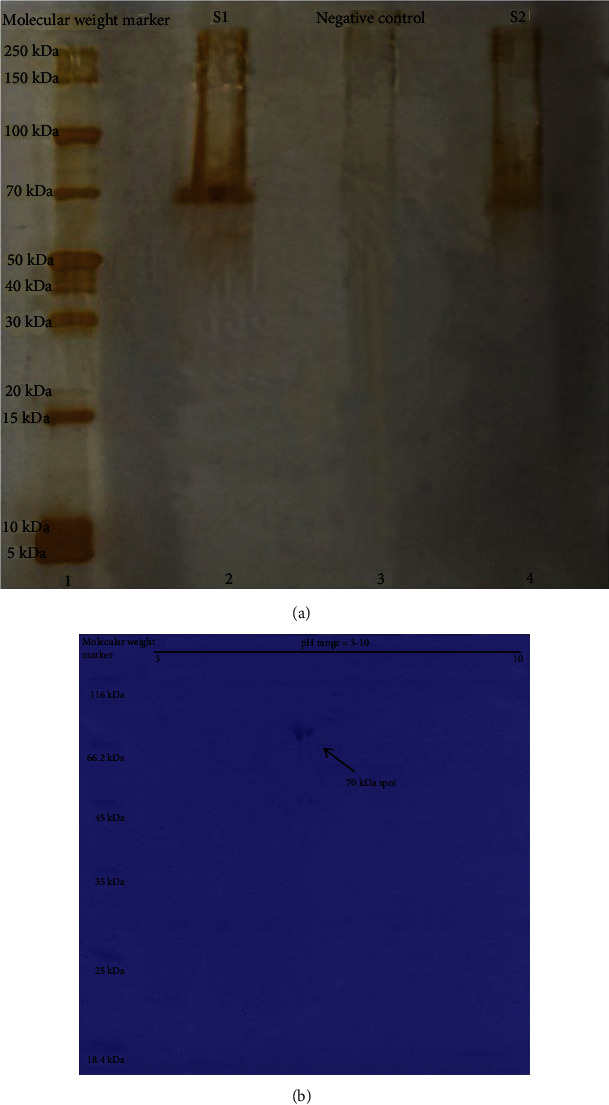
SDS-PAGE and 2-DE pattern of heat-stressed fibroblasts derived proteins. (a) The single protein band with a relative molecular weight of 70 kDa in silver-stained 12% SDS-PAGE which was carried out with two different samples (s1 and s2) prepared from heat-stressed fibroblast supernatants (lanes 2 and 4). This band was not detectable in the culture supernatant of nonstressed cells (lane 3, negative control). Lane 1 is the molecular weight size marker. (b) 2-DE gel of heat-stressed fibroblasts derived protein with Coomassie Blue staining. The arrow shows two protein spots with a relative molecular weight of 70 kDa in the acidic region of the gel.

To obtain an overall view regarding the protein secretion by heat-stressed skin fibroblast, the total protein concentration as well as SDS-PAGE and 2DE pattern was determined in supernatants of stressed cells (*n* = 3) in comparison to unstressed fibroblasts (*n* = 3). Surprisingly, there was a remarkable level of total proteins in supernatants of the stressed cells (mean ± SD: 41.8 ± 14.6 *μ*g/mL) versus an almost undetectable level in the supernatants of unstressed fibroblasts. As the SDS-PAGE pattern shows, only one thick band of 70 kDa protein was observed, repeatedly (Figure 2(a)). This band was separated into two different spots in 2DE gel with molecular weights of 70 kDa and isoelectric points (pI) of 5.3 and 5.5 (Figure 2(b)).

**Figure 3:**
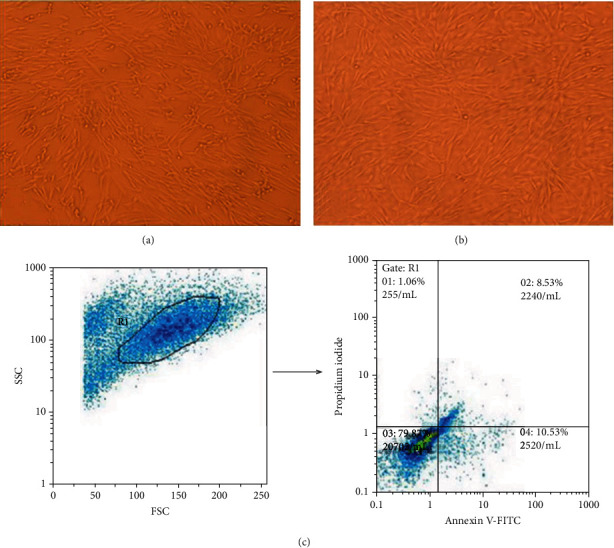
Morphology and cell death pattern of heat-stressed fibroblasts. There is no significant morphological difference between fibroblasts before and after heat shock. (a) Fibroblasts before and (b) after heat shock stress. (c) Forward- and side-scatter plot (left plot) and dot plot graph (quadrant curve) of heat-stressed fibroblasts (right plot). Apoptosis and necrosis cell death pattern were 19% and 1%, respectively (right plot).

To ensure that these proteins were released from live cells, we noticed the cell morphology as well as viability analysis with annexin/PI staining. There was no prominent morphological difference between fibroblasts before and after heat shock, except a little retraction in heat-stressed cells (Figures 3(a) and 3(b)). Moreover, only one percent of cells exhibited the pattern of necrotic cell death, while the majority of heat-stressed fibroblasts remained alive (Figure 3(c)).

### 3.2. Secretory Proteins of Heat-Stressed Fibroblasts Exhibited Stronger Immune Reactivity to the Serum of Normal Subjects versus CAD Patients

**Figure 4:**
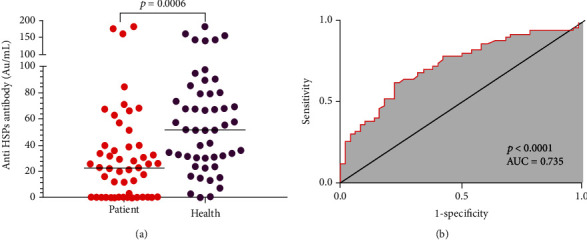
The serum levels of anti-HSP antibody in CAD patients and normal control which shows a diagnostic value of this type of antibody. (a) The anti-HSP IgG was measured by ELISA in serum of CAD patients (*n* = 50) as well as in normal control (*n* = 50). Results are expressed in arbitrary units per milliliter (AU/mL), and the horizontal lines indicated the median. *p* value was equal to 0.0006 when two groups compared with the Mann–Whitney *U* test. (b) The receiver operating characteristic (ROC) curve analysis showed 64% sensitivity and 74% specificity for a cut-off value of 30.5 (AU/mL). The area under the curve (AUC) was 0.74 (*p* < 0.05).

Table 1 shows the demographic variables of CAD patients and normal controls. To study the immune reactivity of fibroblast-derived HSPs, the serum levels of specific IgG antibodies against these proteins were measured by ELISA in serum of CAD patients and normal controls. As it was shown in Figure 4(a), the level of specific anti-HSP IgG in controls was significantly higher than in CAD patients (Table 2).

The lower detection limit of the anti-HSP antibody of the in-house ELISA kit was obtained as 0.5 AU/mL. Besides, the receiver operating characteristic (ROC) curve analysis showed 64% sensitivity and 74% specificity for a cut-off value of 30.5 AU/mL (Figure 4(b)). Then, having this cut-off value, a two-by-two frequency table of specific antibody concentration against HSPs (>30.5 AU/mL or <30.5 AU/mL) versus the presence or absence of coronary atherosclerosis was designed (Table 3). The percentage of anti-HSP IgG negative subjects (<0.5 AU/mL) in patients was higher than in control subjects (30% versus 4%, respectively) (*p* < 0.05). In this sense, the obtained results showed a protective role for anti-HSP antibodies against coronary atherosclerosis. The odds ratio of CAD for the anti-HSP antibody was equal to 5.06 (95%confidence interval (CI) = 2.15‐11.91). The odds ratio was decreased to 4.64 after adjustment of hypertension and gender with anti-HSP antibody (Table 4).

## 4. Conclusion

Despite the notable progress in the treatment and prevention of CAD, atherosclerosis still remains a major cause of mortality at a global level [1]. It may be perceived that bidirectional stress for cardiac cells from the periphery to the heart and vice versa strongly contributes to atherosclerotic CAD [26, 27]. Keep in mind that atherosclerotic lesions are more common at branching sites which are most often exposed to various types of stress such as hyperlipidemia and blood flow disruption [21, 28]. Thus, a more comprehensive view of the cellular stress response to atherosclerosis may open a new horizon into a better understanding of the pathophysiology of the disease as well as the discovery of new diagnostic markers and the development of efficient therapeutic strategies. Although the role of endothelial cells, SMCs, and macrophages in response to ER stress has been well documented, however, the function of fibroblasts in the etiology of atherosclerosis remains unclear. Indeed, targeting this type of cellular stress by using natural compounds has opened a new window in the treatment of atherosclerosis [17]. Hence, the present study provides new insights into the potential role of antifibroblast-derived HSP antibodies in the prevention of coronary atherosclerosis.

Based on the previous reports, HSPs may be produced by skin fibroblasts and keratinocytes in response to the most abundant and potentially harmful environmental stress including UV radiation [29, 30]. HSPs are highly immunogenic molecules that play a critical role in cardiovascular health or disease [31]. HSPs can stimulate the humoral immune responses by which may induce auto-inflammatory conditions such as atherosclerosis [32, 33]. Previous studies have demonstrated a significant increase in circulatory anti-HSP60 antibodies in atherosclerosis patients [34, 35]. Moreover, increased levels of anti-HSP60 antibodies are highly contributing to atherosclerotic cardiovascular disease [36]. Therefore, it is reasonable to presume that the HSPs can be released through skin fibroblasts under physiological conditions which may activate the immune system to produce natural antibodies. The novelty of the current study is the identification of the humoral immune responses of CAD patients and healthy control subjects to the secreted proteins by heat-stressed fibroblasts.

In our study, a remarkable level of protein was determined in heat-stressed fibroblast culture. The flow cytometry analysis showed that nearly one percent of necrotic cell death is characterized by heat-stressed fibroblasts which indicates that proteins could actively be secreted by live cells. Besides, 2DE results demonstrated two spots in the acidic area with close pI and the same molecular weight. Although these spots may be distinguished as stress protein HSP70 and heat shock cognate 71 kDa protein based on bioinformatics search in the ExPASy database, however, immunoblotting data is needed to confirm the conclusion. Interestingly, serum anti-HSP antibody was detectable in nearly 83% of study subjects and was significantly higher in normal controls than in CAD patients. Most likely, genetic variations as well as the polymorphism of immune-related genes contributed to the difference in antibody production between patients with the auto-inflammatory disease and normal controls. Remarkably, promoter polymorphism of the IL-6 gene is significantly associated with anti-HSP60 antibody levels in coronary atherosclerotic patients [37]. Besides, patients with type 2 diabetes have a higher level of anti-HSP60 antibody in association with C-174G polymorphism in the promoter region of the IL-6 gene [38]. Also, sun exposure and the effects of physical activity can be considered other determinants for this status [39]. Furthermore, the reduction of serum anti-HSP antibodies could increase the 5.06-fold risk of CAD, which is reduced to 4.64-folds after adjustment for gender and hypertension. Zhang et al. have indicated that the increasing levels of anti-HSP70 antibody are significantly associated with a reduced risk of acute coronary syndrome (ACS) [40]. Also, Pockley et al. have reported a protective effect of anti-HSP70 antibodies in hypertensive subjects through reduced risk for atherosclerosis [41]. Consequently, we have indicated that fibroblast-derived proteins in response to heat shock may have a potential effect to prevent human coronary atherosclerosis through induction of antibody production. Presumably, these antibodies are able to target ER stress-mediated atherosclerosis; nonetheless, further research is needed to determine the underlying mechanism of antibody-mediated atherosclerosis prevention.

On the other hand, the ROC analysis has revealed that the sensitivity of anti-HSP antibodies with a cut − off < 30.05 AU/mL was 64%. These data indicate that anti-HSP antibody can be considered a diagnostic biomarker for coronary atherosclerosis, with almost the same sensitivity of exercise stress test of 66% [42].

Taken together, human dermal fibroblasts secrete immunogenic proteins with a relative molecular weight of 70-74 kDa in response to heat shock. It may be perceived that an appropriate immunization protocol with HSPs acts as a prophylactic tool in coronary atherosclerosis. Moreover, the antifibroblastic HSP antibody can be considered a novel biomarker for coronary atherosclerosis. This study also showed a relationship between dermal fibroblasts and the health of coronary vessels that could open a new prospect in research for the correlation between fibroblasts and coronary atherosclerosis.

## Figures and Tables

**Table 1:** Demographic variables of CAD patients and normal control.

	CAD patients (*n* = 50)	Normal control (*n* = 50)
Gender (female/male)	19/31	29/21
Age (*mean* ± *SD*) year	62 ± 12.86	60.7 ± 10.39
Hypertensive subjects (*BP*^∗^ ≥ 14 mmHg)/total	15/50	21/50

^∗^Systolic blood pressure.

**Table 2:** The levels of anti-HSP antibody in CAD patients and normal group.

Group	CAD patients (*n* = 50)	Normal controls (*n* = 50)	*p* value
Variable			
Anti-HSP antibody (AU/mL)	31.66 ± 38.01 (22.7)	58.1 ± 40.49 (51.5)	0.0006

Values are shown as mean ± SD (median).

**Table 3:** Two-by-two frequency table of anti-HSP antibody (>30.5/<30.5).

Antibody > 30.5 AU/mL	Positive	Negative	Total
Plaque in coronary arteries			
Positive	(a) 32	(b) 18	50
Negative	(c) 13	(d) 37	50
Total	45	55	100

Odds ratio (confidence interval): 5.06 (2.15-11.91).

**Table 4:** Odds ratio for atherosclerosis in logistic regression.

Variables	Odds ratio	*p* value	95% confidence interval
Hypertension	3.04	0.013	1.18-7.83
Sex	3.34	0.021	1.29-8.63
Anti-HSP antibody	4.64	0.001	1.83-11.67

## Data Availability

The data used to support the findings of this study are available from the corresponding author upon request.
